# Grading of Glioma: combined diagnostic value of amide proton transfer weighted, arterial spin labeling and diffusion weighted magnetic resonance imaging

**DOI:** 10.1186/s12880-020-00450-x

**Published:** 2020-05-14

**Authors:** Xiao-wei Kang, Yi-bin Xi, Ting-ting Liu, Ning Wang, Yuan-qiang Zhu, Xing-rui Wang, Fan Guo

**Affiliations:** 1 Department of Radiology, Xi’an People’s Hospital, Xi’an, ShaanXi China; 2 Department of Radiology, Xijing Hospital, Xi’an, ShaanXi China; 3 Department of Biomedical Engineering, College of Biomedical Engineering & Instrument Science, Zhejiang University, Hangzhou, ShaanXi China; 4 Department of Radiology, The Second Affliated Hospital of Xi’an Medical College, Xi’an, ShaanXi China; 5 Department of Radiology, The Affiliated Hospital of Northwest University (Xi’an No.3 Hospital), Xi’an, ShaanXi China; 6 Key Laboratory of Molecular Imaging of the Chinese Academy of Sciences, Institute of Automation, Chinese Academy of Sciences, Beijing, China

**Keywords:** Glioma, Magnetic resonance imaging, Arterial spin labeling, Amide proton transfer, Apparent diffusion coefficient

## Abstract

**Background:**

To investigate the ability of amide proton transfer (APT) weighted magnetic resonance imaging (MRI), arterial spin labeling (ASL), diffusion weighted imaging (DWI) and the combination for differentiating high-grade gliomas (HGGs) from low-grade gliomas (LGGs).

**Methods:**

Twenty-seven patients including nine LGGs and eighteen HGGs underwent conventional, APT, ASL and DWI MRI with a 3.0-T MR scanner. Histogram analyses was performed and quantitative parameters including mean apparent diffusion coefficient (ADC mean), 20th-percentile ADC (ADC 20th), mean APT (APT mean), 90th-percentile APT (APT 90th), relative mean cerebral blood flow (rCBF mean) and relative 90th-percentile CBF (rCBF 90th) were compared between HGGs and LGGs. The diagnostic performance was evaluated with receiver operating characteristic (ROC) analysis of each parameter and their combination. Correlations were analyzed among the MRI parameters and Ki-67.

**Results:**

The APT values were significantly higher in the HGGs compared to the LGGs (*p* <  0.005), whereas ADC values were significantly lower in HGGs than LGGs (*P* <  0.0001). The ADC 20th and APT mean had higher discrimination abilities compared with other single parameters, with the area under the ROC curve (AUC) of 0.877 and 0.840. Adding ADC parameter, the discrimination ability of APT and rCBF significantly improved. The ADC was negatively correlated with the APT and rCBF value, respectively, while APT value was positively correlated with rCBF value. Significant correlations between ADC values and Ki-67 were also observed.

**Conclusions:**

APT and DWI are valuable in differentiating HGGs from LGGs. The combination of APT, DWI and ASL imaging could improve the ability for discriminating HGGs from LGGs.

## Background

Gliomas are the most common primary tumors of the central nervous system [1]. The ability of discriminating low-grade glioma (LGG) from high-grade glioma (HGG) is of clinical importance as the prognosis and the standard management is different substantially according to the grade. Surgery is an important treatment for HGGs and usually followed by concurrent chemoradiation [2]. Misdiagnosing HGGs as LGGs could lead to insufficient and less aggressive treatment [3]. The current gold standard for gliomas’ diagnosis and therapeutic decision relies on the histopathology, as well as the molecular profile and genetic information [4]. However, the histopathological result depends on biopsy or surgical resection, which is not only invasive, but also affected by the intratumoral histological heterogeneity and sampling erros, which may lead to underestimation of the true grades [3].

In the clinical practice, preoperative magnetic resonance imaging (MRI) contributes to the diagnosis and thus treatment of glioma patients noninvasively. Traditional MRI, such as T2-weighted and contrast enhanced T1-weighted imaging, is conventionally used for diagnosing for characterizing gliomas [5]. HGGs usually show moderate to strong enhancement whereas LGGs showed no or mild enhancement. However, approximately 14–45% HGGs showed no enhancement after contrast agents administration, whereas about 20% enhanced gliomas were histologically proved to be LGGs [6, 7]. Therefore, the accurate grading for gliomas are still challenging with traditional MRI.

Advanced magnetic resonance techniques like dynamic contrast-enhanced (DCE) MRI, susceptibility-weighted imaging (SWI), diffusion-weighted imaging (DWI) and intravoxel incoherent motion (IVIM) have been utilized for glioma grading [8–11]. Amide proton transfer (APT) weighted imaging is a noninvasive emerging molecular MRI technique based on chemical exchange saturation transfer (CEST) between amide protons of proteins and polypeptides and free water protons [12, 13]. Previous study indicated that APT imaging could be used for grading gliomas [3, 4, 14, 15]. It is also valuable for distinguishing pseudoprogression from true progression in gliomas and reflecting treatment response [16, 17]. Arterial spin labeling (ASL) offers non-invasive quantitative measurement of cerebral tissue blood perfusion using magnetically labeled arterial blood water as an endogenous tracer. As one non-contrast perfusion technique, ASL might be one reproducible, quantitative way in daily practice. Multiple studies have reported ASL applied in brain tumor diagnosis and grading [18–20]. Nevertheless, APT and ASL techniques are still limited evaluated in clinical practice and few analysis has been performed with the combination of multiple MRI techniques for grading gliomas [21]. Whether advanced MRI techniques and their combination could accurately reflect the pathological condition of gliomas still needs further verification.

The present study aims to evaluate and compared the diagnostic performance of ASL, APT and DWI and their combination in reflecting the histopathological characteristics and differentiating HGGs from LGGs among gliomas.

## Methods

### Patients

This retrospective study was approved by the institutional review board of Xijing hospital, and the requirement for informed consent was waived. The preoperative MR imaging data of the 27 consecutive patients with gliomas who were identified between September 2015 and June 2018 were analyzed. The histopathologic diagnosis and grades were determined with resection or biopsy specimens according to the WHO criteria by two established neuropathologists, who were blinded to the imaging findings. A total of 27 patients (12 males and 15 females, age range from 29 to 80) including 9 with LGGs (World Health Organization [WHO] grade II and 20 with HGGs (WHO grade III or IV) were identified. The characteristics of patients are described in Table 1.

**Table 1:** Demographics of patients

	LGG (***n*** = 9)	HGG (***n*** = 18)
**Sex (male**: **female)**	3: 6	9: 9
**Age (years)**	48.3 ± 12.2	52.3 ± 13.0
**Histologic feature**		
Glioblastoma (WHO IV)		14
Anaplastic Astrocytomas (WHO III)		2
Anaplastic Oligodendroglioma (WHO III)		1
Anaplastic Oligoastrocytoma (WHO III)		1
Diffuse astrocytomas (WHO II)	6	
Oligodendrogliomas (WHO II)	3	
**Enhancement pattern**		
Markedly heterogeneous enhancement		13
Local Enhancement	2	3
Non-enhancement	7	2
**Number of lesion**		
Single lesion	9	16
Multiple lesion	0	2

Values are mean ± standard deviations

The patients’ histological types of gliomas were as follows: 6 diffuse astrocytomas (3 IDH-mutant, 3 IDH wild-type), 3 oligodendrogliomas (3 IDH-mutant and 1p19q-codeleted), 2 anaplastic astrocytomas (1 IDH wild-type, 1 IDH-mutant), 1 anaplastic oligodendroglioma (IDH-mutant and 1p19q-codeleted), 1 anaplastic oligoastrocytoma (NOS) and 14 glioblastoma multiforme (GBM, 14 IDH wild-type). All patients underwent contrast-enhanced T1- weighted imaging, APT, DW and ASL imaging in their preoperative examinations. The interval between the MR imaging and the surgery was < 2 weeks in all patients. Immunohistochemistry was used to measure the Ki-67 labeling index and 18 patients were recorded (6 LGGs and 12 HGGs).

### MR imaging

MR imaging was performed on a 3.0-T clinical scanner (Discovery MR750, GE Healthcare, Milwaukee, Wisconsin, USA) using an eight-channel phased-array head coil. Sponge padding was used to limit head motion. APT imaging was acquired using the two-dimensional single-shot fast spin echo planner imaging (EPI) and performed using a saturation pulse with a duration of 0.4 s and a saturation power level of B_1_, rms = 2.0μT. For all the patients, the following optimized setting was used: repetition time (TR) = 3000 msec; echo time (TE) = 23 msec; matrix = 128 × 128; slice thickness = 5 mm, field of view (FOV) = 240 × 240 mm^2^; scan time = 3min18s.

ASL was performed with pseudocontinuous labeling, background suppression, and a stack of spiral 3D fast spin-echo imaging sequences using the following acquisition parameters: 512 sampling points on eight spirals, TR = 4632 msec; TE = 10.5 msec; matrix = 128 × 128; slice thickness = 4 mm, field of view (FOV) = 240 × 240 mm^2^; scan time = 4min27s; post-labeling delay = 1525 msec.

The DWI was performed in the axial plane with a single-shot spin-echo echo planar imaging sequence with the following parameters: TR = 3300 msec; TE = 65.8 msec; matrix = 160 × 160 (reconstructed to 256 × 256); slice thickness = 5 mm, FOV = 240 × 240 mm^2^; scan time = 26 s, b values; 0 and 1000 (sec/mm^2^). The apparent diffusion coefficient (ADC) was calculated by mono-exponential fitting with the pair of b-values.

For reference, standard MR images, T1- weighted (TR = 1750 ms; TE = 24 ms; section thickness = 4 mm; inter-slice gap = 0 mm; matrix = 320 × 256); T2-weighted (TR = 3976 ms; TE = 92 ms; inter-slice gap = 1.5 mm; matrix size = 512 × 512); and fluid attenuation inversion recovery (FLAIR, TR = 8400 ms; TE = 145 ms; inter-slice gap = 1.5 mm; matrix size = 160 × 256) and contrast enhanced T1-weighted images (TR = 500 msec, TE = 20 msec, matrix = 256 × 271, 22 slices) were acquired in the axial plane. The FOV size (240 × 240 mm^2^) and the slice thickness (5 mm) are identical in these images except for T1WI.

### Imaging processing

APT, ASL and ADC imaging data were transferred from the MR scanner to an independent personal computer for quantitative data analysis. The data were transferred to an off-line workstation for postprocessing (GE FuncTool software). Data processing was performed by MR specialists with 10 years of clinical experience in MR pulse sequence. For the APT quantification, after water frequency shift correction, magnetization transfer component and the APT (Δω = 3.5 ppm) component, asymmetrical MT ratio (MTR_asym_) analysis was performed according to previous studies [3, 17]. For the region-of-interests (ROIs) were manually segmented on FLAIR and matched to ADC, cerebral blood flow (CBF) and APT maps independently by the two neuroradiologists using the software FireVoxel (CAI2R, New York University, New York, NY), who was blinded to patients’ pathological diagnosis and grading. The areas with necrotic regions, obvious artifacts or signals from a blood vessel, hemorrhage and cystic degeneration were excluded from the segmented area. For ASL, we calculated the relative CBF values (rCBF) by normalizing to the contralateral normal-appearing gray matter. The 20th-percentile ADC (ADC 20th), 90th-percentile APT (APT 90th), 90th-percentile rCBF (rCBF 90th) were derived by the histogram approach in the segmented region [17]. The nth percentile is defined the point at which n% of the voxel values that form the histogram are found to the left. The mean values for the parameters (APT mean, ADC mean and rCBF mean) were also calculated.

### Statistical analysis

All values are expressed as mean ± standard deviation (SD). Interobserver agreement for the tumor rCBF, APT and ADC from the 2 readers was analyzed by calculation of the intraclass correlation coefficient (ICC). ICCs are considered to be excellent if greater than 0.74 [22]. The measurements by the two observers for each patient were averaged for further analysis. The rCBF, APT and ADC values were compared between the LGGs and HGGs groups using Student’s t-test or non-parametric Mann-Whitney U test according to normality test. Correlations between the rCBF, APT signals and ADC values were evaluated with Pearson’s correlation coefficient. The correlation between Ki-67 and those advanced MRI parameters were also evaluated. Receiver operating characteristic (ROC) curve analyses were conducted to evaluate the diagnostic performance of the parameters in differentiating LGGs from HGGs. The area under the ROC curve (AUC) and the optimal cutoff values according to Youden’s index were then calculated. The AUC values < 0.7, 0.7–0.9, and > 0.9 were considered as low, medium and high diagnostic performance, respectively [3]. The integrated discrimination index (IDI), was used to determine the added value [21, 23]. A positive IDI value indicates improved discrimination following the addition of a new parameter. Statistical analyses were performed with a commercially available software package (SPSS, IBM 19, Armonk, NY; Stata software package, version 12.1, StataCorp, College Station, TX). *p* values < 0.05 were considered significant.

## Results

### Characteristics of the study patients

Descriptive statistics regarding the demographic data obtained from the two groups are summarized in Table 1. Supplementary Table 1 shows the ICCs of the measurements by the two observers. Excellent agreement was observed for rCBF, APT and ADC values.

### Differences between LGG and HGG

**Fig. 1:**
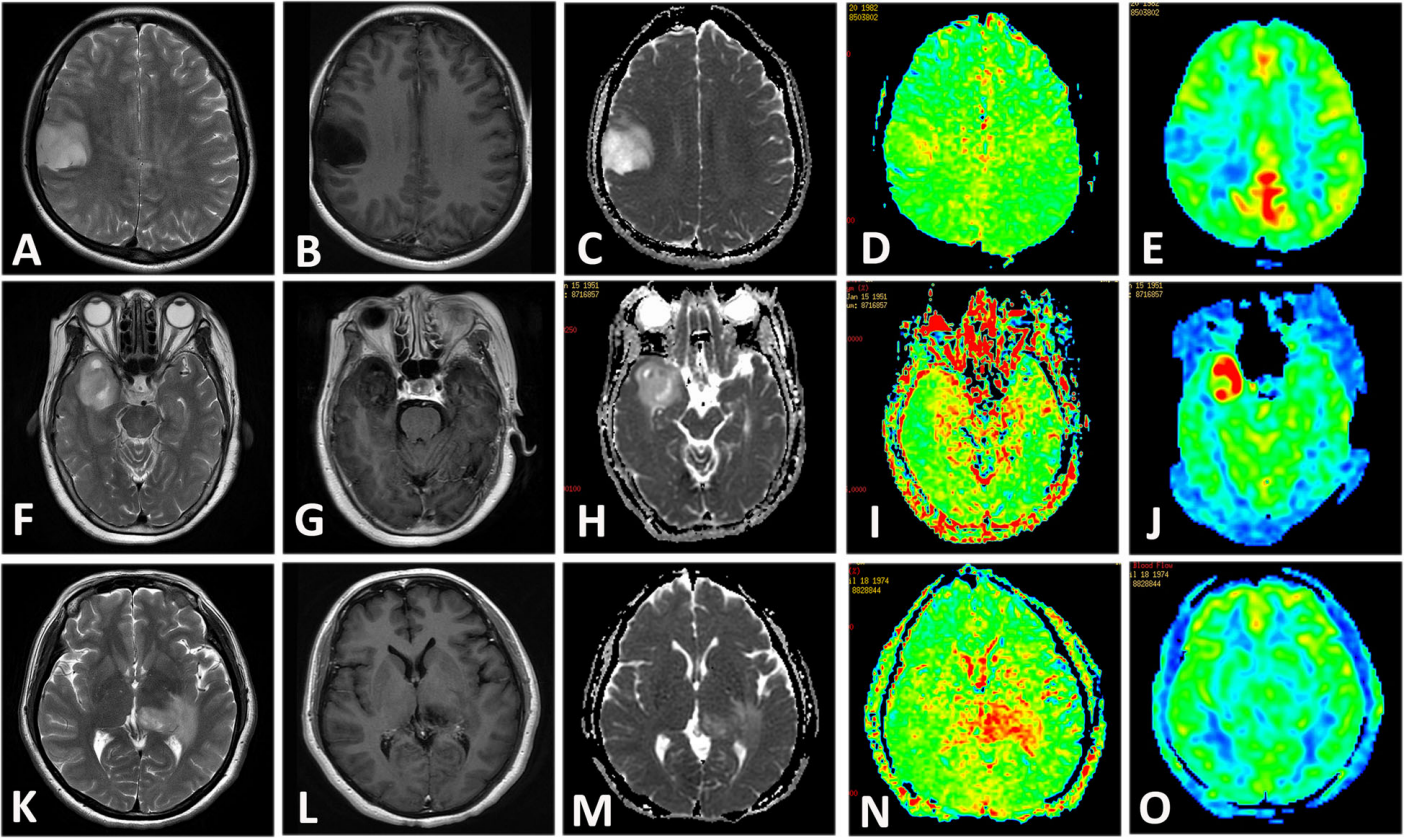
Upper row: A 35-year-old female with diffuse astrocytoma with mutant IDH1 in the right frontal lobe. A transverse T2-weighted image shows a homogeneous hyperintensity area in the right frontal lobe (**a**). A contrast enhanced transverse T1-weighted image shows no enhancement in the tumor (**b**). ADC signal was elevated (**c**). The APT-weighted image shows a mild increase signal in the tumor compared with normal brain tissue (**d**). The CBF map showed low CBF in the tumor (**e**). Middle row: A 66-year-old female patient with diffuse astrocytoma with mutant IDH1 showed a heterogeneously hyperintense area in the right temporal lobe in transverse T2-weighted image (**f**). A contrast enhanced transverse T1-weighted image shows a miner enhancement in the tumor (**g**). ADC signal was slightly elevated (**h**). The APT-weighted image shows mild increase signal in the tumor (**i**). The CBF map showed high CBF in the tumor (**j**). Lower row: A 43-year-old female with a glioblastoma with wide-type IDH1 in the left thalami and temporal lobe. A transverse T2-weighted image shows a heterogeneously hyperintense area in the left temporal lobe (**k**). No obvious local enhancement was observed in postcontrast T1-weighted image (**l**). ADC signal was not uniformly reduced (**m**). APT-weighted image shows high signal in the tumor compared with normal brain tissue (**n**). The CBF map showed no high signal in the tumor (**o**)

As shown in representative images of Fig. 1, the APT signals were higher for HGG. Table 2 summarized the differences in rCBF, ADC values of the tumor entity and APT images between LGGs and HGGs. The APT 90th and the APT mean were significantly higher in HGG group compared to LGG group (*p* < 0.01). And ADC 20th and ADC mean were significantly lower in HGG compared to LGG group (*p* < 0.0001). There was no significant difference of rCBF 90th and rCBF mean between HGG and LGG group (*p* > 0.05).

**Table 2:** Measurements of APT signal and ADC in LGGs and HGGs

	LGG (***n*** = 9)	HGG (***n*** = 18)	***P***
**ADC 20th (×10**^**−3**^**)**	1.25 ± 0.17	0.93 ± 0.18	< 0.0001^*^
**ADC mean (× 10**^**− 3**^**)**	1.41 ± 0.23	1.06 ± 0.19	0.002^†^
**APT mean (%)**	2.06 ± 1.55	3.90 ± 1.20	0.002^*^
**APT 90th (%)**	4.03 ± 2.59	6.46 ± 1.76	0.004^†^
**rCBF 90th**	23.02 ± 38.35	54.01 ± 36.97	0.053
**rCBF mean**	1.06 ± 28.41	22.86 ± 25.64	0.055

^*^P value represents the comparison results of HGG and LGG using the t-test analyzation. ^†^*P* value represents the comparison results of HGG and LGG using non-parametric Mann-Whitney U test. Values are presented as Mean ± standard deviations. *ADC* apparent diffusion coefficient, *APT* amide protein transfer, *ADC mean* mean value of ADC, *ADC 20th* 20th-percentile value of ADC, *APT mean* mean value of APT, *APT 90th* 90th-percentile value of APT, *rCBF 90th* 90th-percentile value or relative cerebral blood flow, *rCBF mean* mean value of cerebral blood flow

### Correlation among the parameters

The ADC values were significantly negatively correlated with the APT values and rCBF values, while the APT values were positively correlated with the rCBF values (Supplementary Table 2, Supplementary Figure). The values of the tumor Ki-67 was correlated with the ADC value (For ADC 20th, *r* = − 0.649, *p* = 0.004; for ADC mean, *r* = − 0.647, *p* = 0.004) but not any of the APT values or rCBF values.

### Diagnostic performance in differentiating HGGs from LGGs

**Fig. 2:**
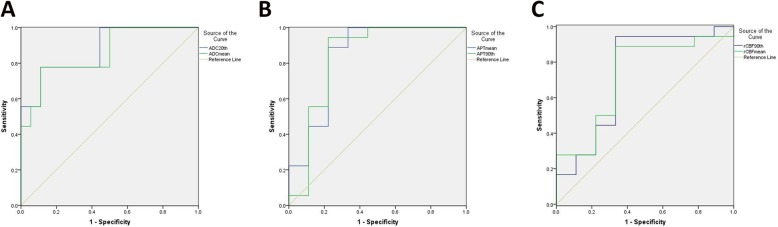
Receiver operating characteristic curve analyses in differentiating LGGs from HGGs of ADC (**a**), APT (**b**) and rCBF (**c**) derived parameters

Table 3 and Fig. 2 summarized the results of the ROC analyses for determining the discriminatory abilities of rCBF, APT and ADC values. Medium diagnostic performance was achieved by all the parameters, with the area under the ROC curve (AUC) of 0.877 for ADC 20th and 0.858 for the ADC mean, and by the APT values, with the AUC of 0.833 for the APT 90th and 0.840 for the APT mean, and by the rCBF values, with the AUC of 0.735 for the ASL 90th and 0.722 for the ASL mean.

**Table 3:** Receiver operating characteristic curve analysis for differentiation of LGGs and HGGs

	AUC	Cutoff Value	Sensitivity (%)	Specificity (%)
**ADC 20th (×10**^**−3**^**)**	0.877	1.13	77.8	88.9
**ADC mean (×10**^**−3**^**)**	0.858	1.27	77.8	88.9
**APT mean (%)**	0.840	2.53	88.9	77.8
**APT 90th (%)**	0.833	4.11	94.4	77.8
**rCBF 90th**	0.735	12.24	94.4	66.7
**rCBF mean**	0.722	−3.61	88.9	66.7

*AUC* area under the receiver operating characteristic curve. *Abbreviations*: *AUC* area under the curve, *ADC mean* mean value of ADC, *ADC 20th* 20th-percentile value of ADC, *APT mean* mean value of APT, *APT 90th* 90th-percentile value of APT, *rCBF 90th* 90th-percentile value or relative cerebral blood flow, *rCBF mean* mean value of cerebral blood flow

Comparing the ROC curves with the highest AUC from each parameters, there were significant differences between ADC 20th and rCBF 90th (*p* = 0.0019), APT mean and rCBF 90th, respectively (*p* = 0.035). Supplementary Table 3 summarized the value of combination parameters. By adding the ADC 20th value to the other two parameters, the AUC increased from 0.840 to 0.907 for APT mean, and 0.735 to 0.883 for rCBF 90th, respectively, and the improvements were significant (*p* = 0.0087, and 0.0011, respectively). Moreover, with the combination of ADC 20th, APT mean and rCBF 90th showed the highest diagnostic performance for distinguishing HGG and LGG, with the AUC of 0.914.

## Discussion

In the current study we investigated the diagnostic performance of APT, DWI and ASL for distinguishing HGGs and LGGs. We found the APT values were higher in the HGGs, whereas ADC values were significantly lower in HGGs. We also observed that histogram values of ADC and APT were compatible for glioma grading. Moreover, ADC parameter improved the performance of APT and rCBF for discriminating HGGs from LGGs.

Although currently the diagnosis and therapeutic decision of gliomas relies on histopathology, molecular profile and genetic information as gold standard, tumor heterogeneity could cause an underestimation of true grading because of the sampling error in biopsies [21]. In the clinical practice, traditional MRI protocols sometimes are inadequate for accurate grading [4]. For example, as the gliomas enhancement might be caused by disruption of the blood-brain barrier instead of neovascularization, the degree of contrast enhancement of gliomas is not constantly dependable for distinguishing LGGs from HGGs [3, 7]. Advanced imaging techniques including DWI, perfusion-weighted imaging (PWI) and proton MR spectroscopy could provide additional information for grading tumors and have been increasing practiced clinically [10, 24, 25]. However, inconsistent results or overlap in measured values usually resulted from different methods and different studies. Novel imaging methods that complement each other, thus improving grading accuracy, is still urgently needed.

In our study, we first observed that APT signal intensities were higher for HGGs than LGGs. APT imaging is a novel molecular MRI method and provides information predominantly based on the amide protons in cellular proteins and peptides in the intracellular space and extracellular space [3]. Higher peptide or protein concentration could result from higher cell density in HGGs [26, 27]. Previous studies have demonstrated that APT signal intensity was positively correlated with the glioma grade [4, 26]. Similarly, our results showed higher APT signal intensity in HGGs than LGGs, indicating different mobile protein and peptide concentrations in the brain tumor according to the glioma grade [4, 28]. On the other hand, we also observed that lower ADC intensities in HGGs compared with LGGs. It was consistent with pervious published paper that ADC was correlated with glioma grade and reflect tumor cellularity and water content in interstitial space [29]. Bulakbasi et al. proved that ADC is helpful for discriminating HGGs from LGGs [10]. Some controversial result showed that ADC did not differ between the HGGs and LGGs [3, 30]. The nth percentile is the point at which n % of the voxel values that form the histogram are found to the left. The 20% histogram cutoffs for the ADC and 90% histogram cutoffs for the rCBF were derived and proved with high AUC. This type of parameter can sometimes be more effective than the maximum or mean values, as it is less influenced by random statistical fluctuation [17, 31]. This may indicate that 20th–percentile ADC and 90th–percentile rCBF might better reflect the content of boundary area. However, we observed there was no significant difference between LGG and HGG in rCBF although there was the tendency of a slightly higher value in HGG than in LGG. Our result was partially consistent with previous studies [32, 33]. ASL might be one useful way to distinguish HGGs and LGGs but may not be reliable enough. Further larger-scale studies are necessary to further study the utility of ASL for grading glioma.

Secondly, we found a negative correlation between ADC value and Ki-67, which is consistent with previous study [34]. We did not observe correlation between APT or rCBF and Ki-67. This result is controversial from previous study which indicates APT signal intensity is associated with the activity of tumor cells proliferation of tumor cells [4, 35]. The inconsistent correlation results may be due to the sample size or the different locations between histology and the ROIs of APT imaging. The matching between MRI and histopathology is still challenging.

Our results showed that the ADC improved the diagnostic ability when added to the APT signal or rCBF. As DWI could be performed within short period of time, it is currently widely used in the daily practice. Our study may indicate that DWI should be practice more frequently with routine conventional MRI. Although ROC suggested that APT, rCBF and ADC had a comparable medium diagnostic value for LGGs compared with HGGs, we proved that the highest diagnostic value was obtained when 20th-percentile ADC, mean value of APT and 90th-percentile rCBF were combined. Previous study indicated that APT imaging increased the accuracy of regional cerebral blood volume for differentiating contrast enhanced LGG from HGG [36]. Choi et al. proved that APT imaging added value to the ADC for discriminating between LGG and HGG [21]. This may indicate that combination of more neuronal imaging could offer more diagnostic information, thus improving the diagnostic performance. As none of APT, ASL or ADC requires an exogenous contrast agent, our results imply that those techniques are therefore powerful diagnostic tools for refining glioma histopathology and grades especially when the patients’ condition is not recommended with contrast agent injection.

Our study has several limitations. First, the cohort is relatively small, especially that of the LGGs, and there are many subtypes of gliomas included in the study. Larger sample sizes are needed in the future, as well as a validation cohort. Second, IDH genotypes were ignored in the included patients and the genotypes could affect the tumor angiogenesis, therefore leading to the low diagnostic performance for ASL. Thirdly, single slice acquisition was applied in the APT imaging sequence as the total time for clinical patient scans was limited. Therefore, it was possible that the entire tumor was not covered properly. Finally, our conclusions need further collection of patients for validation test.

## Conclusions

We showed that DWI and APT imaging are useful for glioma grading. Moreover, ADC combined with ADC and rCBF value showed strong ability for discriminating HGGs from LGGs. Our result indicated that ADC, APT as well as ASL imaging may serve as a powerful technique of grading gliomas, thus facilitate effective diagnose and therapy for glioma patients.

## Supplementary information

**Additional file 1: Supplementary Table 1.** Inter-observer agreement. **Supplementary Table 2.** Correlation between Ki-67 and the parameters. **Supplementary Table 3.** Comparison of the area under the receiver-operating characteristic curve of the combinations of MRI parameters. **Supplementary Figure.** Correlation between Ki-67 and the MRI parameters.

## Data Availability

The datasets used and analysed during the current study are available from the corresponding author on reasonable request.

## Abbreviations

APT: Amide proton transfer
MRI: Magnetic resonance imaging
ASL: Arterial spin labeling
DWI: Diffusion weighted imaging
HGG: High-grade glioma
LGG: Low-grade glioma
ADC: Apparent diffusion coefficient
ADC 20th: 20th-percentile ADC
APT 90th: 90th-percentile APT
rCBF mean: relative mean cerebral blood flow
rCBF 90th: relative 90th-percentile CBF
ROC: Receiver operating characteristic
AUC: Area under the ROC curve
DCE: Dynamic contrast-enhanced
SWI: Susceptibility-weighted imaging
CEST: Endogenous chemical exchange saturation transfer
ICC: Intraclass correlation coefficient
