# Supplementary material for: Grading of Glioma: combined diagnostic value of amide proton transfer weighted, arterial spin labeling and diffusion weighted magnetic resonance imaging
Source: BMC Med Imaging. 2020 May 14;20:50. doi: 10.1186/s12880-020-00450-x (PMC7227252; doi:10.1186/s12880-020-00450-x)
Supplement: Supplementary file 1 — Additional file 1: Supplementary Table 1. Inter-observer agreement. Supplementary Table 2. Correlation between Ki-67 and the parameters. Supplementary Table 3. Comparison of the area under the receiver-operating characteristic curve of the combinations of MRI parameters. Supplementary Figure. Correlation between Ki-67 and the MRI parameters. [file 12880_2020_450_MOESM1_ESM.zip › Supplymentary TableR2.docx]

**Supplementary Table 1 Inter-observer agreement**

|  | ICC |
| --- | --- |
| ADC 20^th^ (×10^-3^) | 0.840 |
| ADC mean (×10^-3^) | 0.923 |
| APT mean (%) | 0.956 |
| APT 90^th^ (%) | 0.963 |
| rCBF 90^th^ | 0.955 |
| rCBF mean | 0.898 |

ICC, intraclass correlation coefficient; ADC, apparent diffusion coefficient; APT, amide protein transfer; ADC mean, mean value of ADC; ADC 20^th^, 20th-percentile value of ADC; APT mean, mean value of APT; APT 90^th^, 90th-percentile value of APT; rCBF 90^th^ , 90^th^-percentile value or relative cerebral blood flow; rCBF mean, mean value of cerebral blood flow.

**Supplementary Table 2 Correlation between Ki-67 and the parameters**

|  |  | **ADC 20^th^** | **ADC mean** | **APT mean** | **APT 90^th^** | **rCBF 90^th^** | **rCBF mean** |
| --- | --- | --- | --- | --- | --- | --- | --- |
| **Ki-67** | **r** | -0.649 | -0.647 | 0.176 | 0.121 | 0.275 | 0.254 |
|  | **P** | **0.004^*^** | **0.004^*^** | 0.484 | 0.632 | 0.270 | 0.310 |
| **ADC 20^th^** | **r** | / | 0.989 | -0.558 | -0.432 | -0.440 | -0.402 |
|  | **P** | / | < 0.0001 | **0.002^*^** | **0.024^*^** | **0.022^*^** | **0.038^*^** |
| **ADC mean** | **r** |  | / | -0.545 | -0.410 | -0.455 | -0.424 |
|  | **P** |  | / | **0.003^*^** | **0.034^*^** | **0.017^*^** | **0.027^*^** |
| **APT mean** | **r** |  |  | / | 0.870 | 0.625 | 0.665 |
|  | **P** |  |  | / | < 0.0001 | **< 0.0001^*^** | **< 0.0001^*^** |
| **APT 90^th^** | **r** |  |  |  | / | 0.449 | 0.466 |
|  | **P** |  |  |  | / | **0.019^*^** | **0.014^*^** |
| **rCBF 90^th^** | **r** |  |  |  |  | / | 0.966 |
|  | **P** |  |  |  |  | / | < 0.0001 |
| **rCBF mean** | **r** |  |  |  |  |  | / |
|  | **P** |  |  |  |  |  | / |

P value represents the comparison results of HGG and LGG using Pearson correlation. ADC, apparent diffusion coefficient; APT, amide protein transfer; ADC mean, mean value of ADC; ADC 20^th^, 20th-percentile value of ADC; APT mean, mean value of APT; APT 90^th^, 90th-percentile value of APT; rCBF 90^th^ , 90^th^-percentile value or relative cerebral blood flow; rCBF mean, mean value of cerebral blood flow.

**Supplementary Table 3 Comparison of the area under the receiver-operating characteristic curve of the combinations of MRI parameters**

| Parameters | AUC | 95% CI | P value for IDI |
| --- | --- | --- | --- |
| ADC 20^th^ + APT mean | 0.907 | 0.785-1.000 | 0.0087 vs. APT mean |
| ADC 20^th^ + rCBF 90^th^ | 0.883 | 0.749-1.000 | 0.0011 vs. rCBF 90^th^  0.0084 vs. APT mean + rCBF 90^th^ |
| APT mean + rCBF 90^th^ | 0.815 | 0.610-1.000 |  |
| ADC 20^th^ + APT mean + rCBF 90^th^ | 0.914 | 0.794-1.000 | 0.0006 vs. APT mean + rCBF 90^th^ |

ADC, apparent diffusion coefficient; APT, amide protein transfer; ADC mean, mean value of ADC; ADC 20^th^, 20th-percentile value of ADC; APT mean, mean value of APT; APT 90^th^, 90th-percentile value of APT; rCBF 90^th^ , 90^th^-percentile value or relative cerebral blood flow; rCBF mean, mean value of cerebral blood flow.

**Supplementary Figure. Correlation between Ki-67, ADC mean, ADC 20^th^, APT mean, APT 90^th^, rCBF 90^th^ and rCBF mean.** ADC, apparent diffusion coefficient; APT, amide protein transfer; ADC mean, mean value of ADC; ADC 20^th^, 20th-percentile value of ADC; APT mean, mean value of APT; APT 90^th^, 90th-percentile value of APT; rCBF 90^th^ , 90^th^-percentile value or relative cerebral blood flow; rCBF mean, mean value of cerebral blood flow.
