# Supplementary figures and images for: Grading of Glioma: combined diagnostic value of amide proton transfer weighted, arterial spin labeling and diffusion weighted magnetic resonance imaging
Source: BMC Med Imaging. 2020 May 14;20:50. doi: 10.1186/s12880-020-00450-x (PMC7227252; doi:10.1186/s12880-020-00450-x)

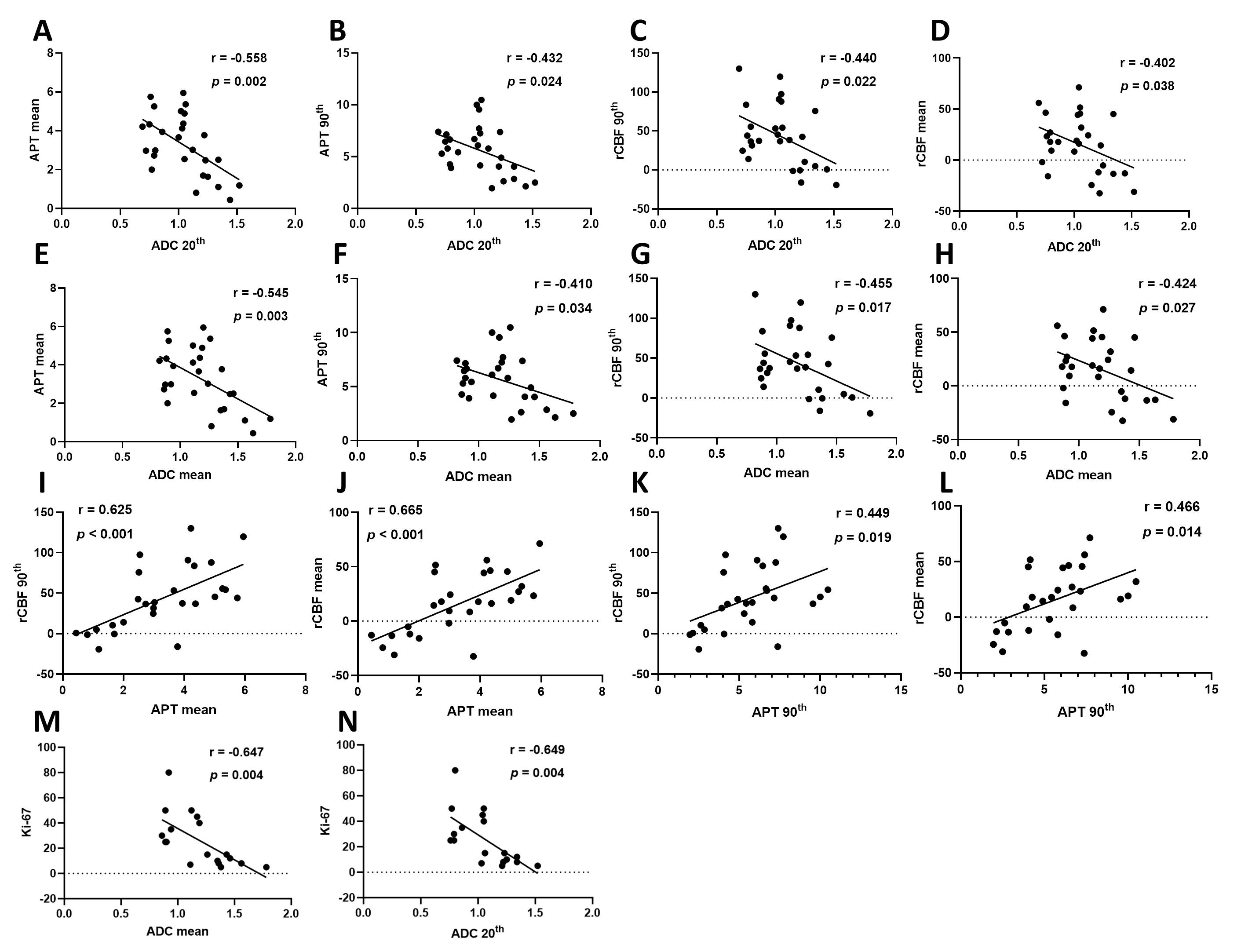

Supplement: Supplementary file 1 — Additional file 1: Supplementary Table 1. Inter-observer agreement. Supplementary Table 2. Correlation between Ki-67 and the parameters. Supplementary Table 3. Comparison of the area under the receiver-operating characteristic curve of the combinations of MRI parameters. Supplementary Figure. Correlation between Ki-67 and the MRI parameters. [file 12880_2020_450_MOESM1_ESM.zip › Supplementary FigureR2.jpg]
